# Chronic Inhibition of FAAH Reduces Depressive-Like Behavior and Improves Dentate Gyrus Proliferation after Chronic Unpredictable Stress Exposure

**DOI:** 10.1155/2021/6651492

**Published:** 2021-03-24

**Authors:** A. R. Tejeda-Martínez, J. M. Viveros-Paredes, G. V. Hidalgo-Franco, E. Pardo-González, V. Chaparro-Huerta, R. E. González-Castañeda, M. E. Flores-Soto

**Affiliations:** ^1^Laboratorio de Neurobiología Celular y Molecular, División de Neurociencias, Centro de Investigación Biomédica de Occidente (CIBO), Instituto Mexicano del Seguro Social, 44340, Mexico; ^2^Laboratorio de Investigación y Desarrollo Farmacéutico, Departamento de Farmacología, Centro Universitario de Ciencias Exactas e Ingenierías, Universidad de Guadalajara, 44430, Mexico; ^3^Laboratorio de Microscopia de alta resolución, Departamento de Neurociencias, Centro Universitario de Ciencias de la Salud, Universidad de Guadalajara, 44340, Mexico

## Abstract

Symptoms of depressive disorders such as anhedonia and despair can be a product of an aberrant adaptation to stress conditions. Chronic unpredictable stress model (CUS) can generate an increase in the activity of the hypothalamic-pituitary-adrenal axis (HPA) and induce a reduction of neurotrophin signaling and the proliferation of neural progenitors in the adult dentate gyrus, together with increased oxidative stress. Levels of the endocannabinoid anandamide (AEA) seem to affect these depression-by-stress-related features and could be modulated by fatty acid amide hydrolase (FAAH). We aimed to evaluate the effects of FAAH inhibitor, URB597, on depressive-like behavior and neural proliferation of mice subjected to a model of CUS. URB597 was administered intraperitoneally at a dose of 0.2 mg/kg for 14 days after CUS. Depressive-like behaviors, anhedonia, and despair were evaluated in the splash and forced swimming tests, respectively. Alterations at the HPA axis level were analyzed using the relative weight of adrenal glands and serum corticosterone levels. Oxidative stress and brain-derived neurotrophic factor (BDNF) were also evaluated. Fluorescence immunohistochemistry tests were performed for the immunoreactivity of BrdU and Sox2 colabeling for comparison of neural precursors. The administration of URB597 was able to reverse the depressive-like behavior generated in mice after the model. Likewise, other physiological responses associated with CUS were reduced in the treated group, among them, increase in the relative weight of the adrenal glands, increased oxidative stress, and decreased BDNF and number of neural precursors. Most of these auspicious responses to enzyme inhibitor administration were blocked by employing a cannabinoid receptor antagonist. In conclusion, the chronic inhibition of FAAH generated an antidepressant effect, promoting neural progenitor proliferation and BDNF expression, while reducing adrenal gland weight and oxidative stress in mice under the CUS model.

## 1. Introduction

Clinical depression is widespread and debilitating; it is characterized by the presence of symptoms such as anhedonia and despair [1]. In recent WHO reports, more than 300 million people (4.4% of the world population) suffer from depression globally; therefore, it could be a guiding factor of disease burden by 2030. Various experimental pieces of evidence have shown that depressive symptoms may be the outcome of an aberrant adaptation to chronic stress conditions, which increases the activity of the hypothalamic-pituitary-adrenal (HPA) axis [2, 3] leading to adrenal impairment that includes hypertrophy of the gland and exacerbated reactivity to corticosterone [4]. These circumstances, along with the lack of serotonin, have a repercussion dampening neurotrophic factor levels, such as those of the brain-derived neurotrophic factor (BDNF) and neurogenesis. Therefore, a significant reduction in the proliferation of neural precursors in the dentate gyrus (DG) has been linked to depression-related phenotypes [5]. In this sense, depressed adults without any antidepressant treatment had fewer granular neurons in the anterior DG compared to healthy controls [6], which is consistent with the findings of reduced hippocampal volume observed in patients with major depression [7]. Moreover, raising glucocorticoid levels in humans induces reactive oxygen species and nitrogen production and increases oxidative stress, which leads to increased lipid peroxidation [8]. Currently, the pharmacological gamma to treat depression includes monoamine oxidase inhibitors, selective serotonin reuptake inhibitors, serotonin-norepinephrine reuptake inhibitors, and tricyclic antidepressants [9]. However, these antidepressant treatments are not universally effective [10], and many of them result in severe side effects, such as cognitive impairment, sexual dysfunction, sleep disturbance, and urinary retention, thus leading to poor therapeutic compliance [11]. Therefore, we see an urgent need to develop more effective and safer pharmacological treatments for depression through the modulation of various neurotransmission systems such as the endocannabinoid system. This system consists of its specific receptors, type 1 and type 2 cannabinoid receptors (CB1R and CB2R, respectively); its endogenous ligands, 2-arachidonoylglycerol (2-AG) and anandamide (AEA); its recapture system; and the enzymes that participate in the synthesis: N-acyltransferase and phospholipase D, and degradation: fatty acid amide hydrolase (FAAH) and monoacyl-glycerol lipase (MAGL) of endogenous endocannabinoids [12]. In this sense, it has been shown that the endocannabinoid system plays a central role in certain neuropsychiatric disorders, particularly those involving affective disturbances such as anxiety and depression [13]. Several CB1R/CB2R agonists have been used to explore the endocannabinoid system as a therapeutic target in depression. The antidepressant effects of these compounds in ameliorating the disorders of the HPA axis and reversing depressive-like behaviors have been demonstrated in animal models [14]. The use of knockout mice for CB1R has also been postulated as a genetic model of depression, where the mutated mice have shown increased depressive behavior, such as increased immobility in the forced swimming test (FST) versus their wild-type counterparts [15]. There is evidence for CB1R-mediated hippocampal neurogenesis *in vivo* in C57 mice subjected to the *CB1 synthetic agonist* arachidonyl-2-chloroethylamide administration [16]. For his part, stimulation of CB2R has been capable of generating neural progenitor cell proliferation in healthy mouse hippocampus through the activation of the mTOR1 signaling pathway [17]. These pieces of evidence underline the importance of the signaling of these receptors; however, direct stimulation of a receptor by a cannabinoid agonist makes the action of the compound on the receptor less robust than indirect stimulation and causes this strategy to be prone to side effects. Hence, inhibition of FAAH and MAGL that indirectly increases the excitability of the endocannabinoid system by reducing the hydrolysis of endocannabinoids could be a more promising therapeutic approach against depressive disorders [18]. The inhibition of FAAH has been described as a strategy capable of augmentation of the brain-derived neurotrophic factor (BDNF), in rats with genetic susceptibility to present depressive behaviors that were also reversed in these subjects [19]. Inhibition of FAAH was able to increase the firing rate of serotoninergic neurons on the dorsal raphe and exerted a reduction in depressive behaviors, similar to those exerted by citalopram and imipramine in healthy rats [20]. Nevertheless, the effects of the inhibition of FAAH need to be further analyzed in other models that mimic the features of depression like the chronic unpredictable stress model (CUS) to understand their role in neurogenesis and behavior. The model of CUS has demonstrated to have an impact on the depressive-like behaviors, accompanied by a series of physiological changes, such as an augmentation of adrenal gland weight, corticosterone, and oxidative stress, along with BDNF and neurogenesis diminishment making it a reliable tool to this aim [21, 22]. This study was aimed at evaluating if the effects of chronic inhibition of FAAH could be capable of restoring neurogenesis and behavioral impairment in mice subjected to a depression model by CUS.

## 2. Materials and Methods

### 2.1. Animal Preparation

For this study, 87 male C57BL/6J mice were used, each weighing 25–30 g, obtained from Harlan Laboratories (Mexico City). Mice were kept on a 12:00 h light-dark cycle, with food and water available ad libitum. All experimental procedures were consistent with ethical policies stipulated by the Ethical Research Committee of Centro de Investigación Biomédica de Occidente (R-2017-1305-6) and were realized according to the official Mexican Norms NOM-062-ZOO-1999 and NOM-033-ZOO-1995 as well as National Institutes of Health guide for the care and use of laboratory animals (NIH Publications No. 8023, revised 1978).

### 2.2. Drug Administration

This study was designed to evaluate the effects of chronic inhibition of FAAH in the CUS model of depression. Mice were randomly distributed in 6 groups (*n* = 12 per group) as follows: the control group received the vehicle without exposition to CUS. The URB597 group received URB597 (a proven selective FAAH inhibitor by Piomelli and collaborators [23]) once per day for 14 days (0.2 mg/kg, i.p.; Sigma-Aldrich). The CUS group received the vehicle after exposition to the model. CUS+URB597 received the same pharmacological treatment as the URB597 group starting once CUS was finished. Finally, the CUS+RIM+URB597 group received rimonabant (RIM) (an antagonist for CB1R, 1 mg/kg, i.p.; Tocris) once per day 30 minutes before each URB597 administration, and these started once CUS was finished.

### 2.3. Chronic Unpredictable Stress Model (CUS)

The CUS model was used to simulate the behavioral and pathophysiological conditions of depression [24]. This model is a modification of the Patterson technique [25], which consists in the application of the following stressors: strobe light in a dark room, bed sawdust wet, water deprivation, deprivation of food and water, with an inclination of the cage at 45° with respect to the horizontal axis, overcrowding (mice were placed 4 per box in a space of 15 × 10 × 5 cm), and change from light to dark every 15 min. These stressors were randomly carried (so that the animals could not predict the occurrence of stimulation) out once a day for a period of 2 hours each for a total of 14 hours per day, for 14 days, during the light phase and the 2 last hours of the dark phase.

### 2.4. Assessment of Plasma Corticosterone Concentration

We quantified corticosterone concentration in mice that were not exposed and exposed to the model for half of the total period (7 days) and the total period of the CUS model (14 days) to validate its effects. Animals were decapitated, and blood serum was collected after allowing clot and centrifuged at 1000 × g for 15 minutes and then stored at −80°C until assay. The concentration of corticosterone in serum was measured using a commercial enzyme-linked immunosorbent assay (ELISA) kit following the manufacturer's instructions (EA66, Oxford Biomedical Research, CUSABIO, Wuhan, China). A linear regression equation of the standard curve was set up based on the concentrations of standards. The optical density (OD) of the solution was quantified with a microplate spectrophotometer at 450 nm. This test was conducted using 15 mice (5 per cohort) while the rest of the animals were assigned to the groups previously described in the text.

### 2.5. Thiobarbituric Acid-Reactive Substance (TBARS) Assay

Six animals per group were decapitated, and hippocampi were collected, immediately placed on dry ice, and stored at −80°C until assay. Samples were homogenized in a standard lysis buffer (100 mM Tris, pH 7.4, 150 mM NaCl, 1 mM EGTA, 1 Mm EDTA, 1% Triton X-100, and sodium deoxycholate 0.5%) and protease inhibitor solution (Complete™; Sigma-Aldrich, 05 056 489 001) and centrifuged at 13,000 rpm for 30 min. The levels of lipid peroxidation were measured using the TBARS assay kit FR22 (Oxford Biomedical Research) according to the manufacturer's instructions. In this method, the peroxide lipids converge in the formation of malondialdehyde (MDA), the final product of lipid peroxidation; this molecule reacts with 2-thiobarbituric acid (TBA) to form Schiff bases. These complexes exhibit colors whose concentrations can be determined spectrophotometrically at 586 mm. The results are expressed in *μ*M of malondialdehyde (MDA).

### 2.6. Behavioral Tests

**Figure 1:**
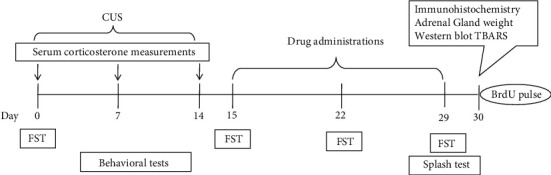
Schematic representation of the experimental design. On day 0 of the experimental protocol, mice performed the pretest for forced swimming test (FST) and habituation for splash test, then were subjected to the CUS protocol (corticosterone measurements were made on a separate group of mice). On day 15, there was another FST evaluation before treatment administration and this was repeated on days 22 and 29 (last day splash test was evaluated).

For the assessment of the behavioral aspects, the splash and forced swimming tests (FST) were used to measure depressive-like behaviors (Figure 1).

#### 2.6.1. Splash Test

The test session consisted of applying a 10% saccharose solution with a sprinkler (100 *μ*L) on the lower back of the animal and then placing it in an acrylic cylinder (20 cm in diameter and 30 cm high); his movements were then video recorded. Grooming in the area where the solution was placed was measured both in its latency and total time, as well as the average duration per grooming and the number of grooming during the session [26]. All these parameters were defined as a measure of anhedonia of the experimental subject. In other words, the greater and the more the grooming behavior and the lower the latency, the lesser the anhedonia shown by mice [27]. Mice had previous habituation of 20 minutes without the stimulus in the place where the video recording was performed.

#### 2.6.2. Forced Swimming Test (FST)

The FST was carried out following the classical protocol from Porsolt et al. [28], also known as the behavioral despair test, which is based on a rodent's response to the threat of drowning, the results of which have been interpreted as a measure of susceptibility to negative mood. Mice were placed in clear glass cylinders (30 cm tall × 20 cm diameter) filled with water (25°C), approximately 15 cm deep, to prevent their tails from touching the bottom. The first session (pretest) was conducted in order to condition the mouse for the impossibility of escaping for subsequent evaluation of despair. Twenty-four hours later, the mouse was again subjected to the test for 5 min to evaluate the total time spent immobile during the whole test (despair), defined as the absence of movement, except the required movement to keep the head above water [29]. Test sessions were recorded with a video camera, and the duration of immobility was scored by 3 different experimental evaluators. All mice followed the same experimental chronogram starting at day 0, ending with a final behavioral assessment on day 29.

### 2.7. Western Blot Analysis

The previously described homogenates were used for this assay. The protein concentration was determined using the Lowry method. Samples (30 *μ*g total protein) were separated by SDS-polyacrylamide gel electrophoresis and then transferred onto a PVDF membrane. The membrane was blocked with 5% skim milk in Tris-buffered saline and then incubated at 4°C overnight with the respective primary antibodies for anti-BDNF antibody (1 : 1000, AB108319 Abcam) or anti-*β*-actin antibody (1 : 5000, MA1-140 ThermoFisher). After washing with Tris-buffered saline with Tween 20 (TBST), the membranes were incubated for 2 h with biotinylated goat anti-rabbit IgG (1 : 1,000, BA 1000; Vector Laboratories) as a secondary antibody. After five washes (PBS-Tween-20, 0.05%), the membranes were incubated with the ABC Elite kit (PK6100; Vector Laboratories) for 1 h, and subsequently, the membranes were developed with diaminobenzidine (D5905; Sigma). Protein expression was assessed using free-to-use ImageJ software (Wayne Rasband, National Institutes of Health, USA, version 1.51j8), and the data obtained were normalized to the area per line as described suitable for individual protein analysis by Bass and collaborators [30], before using the corresponding expression of *β*-actin as an internal control in each sample. Data were reported as a percentage of normalized area relative to control and presented as the mean of at least six independent experiments.

### 2.8. Immunohistochemical Determination of Neuronal Precursor Proliferation by Fluorescence of BrdU+/Sox2+ Cells

To this end, 5-bromo-2′-deoxyuridine (BrdU) (B5002; Sigma-Aldrich) was administered two hours before sacrifice at a dose of 100 mg/kg i.p. in saline solution. Immediately, the mice were anesthetized with ketamine (100 mg/kg., i.p.) and xylazine (15 mg/kg, i.p.) and perfused intracardially with a 0.1 M PBS (phosphate-buffered saline) solution followed by 4% paraformaldehyde (PFA) solution in PBS. After perfusion, the animal's brains were removed, left in fresh PFA fixative for 24 h, and washed 3 times with 0.1 M PBS, and finally, coronal vibratome slices (35 *μ*m, Leica VT1000E; Leica Microsystems, Wetzlar, Germany) of the region of the DG (bregma 1.70 to 0.14 mm) according to Paxinos and Franklin [31] were obtained. From each brain, 6 tissues were collected per individual, with a distance between each slice of 175 *μ*m. Tissue samples were treated with 2 N HCl for 10 minutes at 37°C, followed by 0.1 M borate buffer at pH 8.5 for 10 minutes. Brain sections were rinsed four times in 0.1 M PBS and incubated in the blocking solution (PBS, 0.1 M, Triton X-100, 0.03%, and 10% fetal bovine serum) for 50 minutes. Subsequently, the free-floating samples were incubated overnight with primary antibody rat IgG anti-BrdU, a marker for cell proliferation (1 : 500; Bio-Rad, Kidlington, UK; Cat# OBT0030), and anti-Sox2, a marker for neural stem cells (1 : 500; Millipore, Billerica, MA, USA; Cat# AB5603), at 4°C. Sections were then rinsed 4× with 0.1 M PBS and incubated with the same blocking solution containing the conjugated secondary antibodies (1 : 1000 Alexa Flour 488 anti-rat Cat# A-21208 and 1 : 1000 Alexa Flour 594 anti-rabbit Cat # R37117, ThermoFisher, Waltham, MA, USA) for 1 hour at room temperature. After rinsing (4× with 0.1 M PBS), nuclear staining was done with DAPI (Abcam, Cambridge, MA, USA; Cat# ab104139). The sections were washed with 0.1 M PBS and mounted on glass slides and covered with Vectashield mounting media (Vector Laboratories, Burlingame, CA, USA; Cat# H-1000). All slices from each mouse were counted to a total of 12 DG per mouse (6 per hemisphere) ranging from -0.94 to -2.8 mm relative to the bregma. Every DG was analyzed entirely counting all positive cells following the subgranular zone, taking in a count from 1 field to 3 depending on the anteroposterior exact location of the slice. The slices were analyzed in an Axioskop Zeiss microscope, and photomicrographs were taken with an OLYMPUS DP70 camera. Then, the channels were separated and merged with the ImageJ program to count all merged marks per subgranular zone of DG per slice.

### 2.9. Relative Weight of the Adrenal Glands

Adrenal glands were extracted after the fixation process, which were obtained from the retroperitoneal connective tissue located on the kidneys, removing the adipose tissue, and then weighed on an analytical balance. Fixed tissues were dried for 5 min before the measurement of their weight to avoid errors in the weighing of the remnants of the fixing solution. The relative adrenal gland weights (mg per pair of dry glands/total body weight of each mouse in g) were calculated as described previously [32].

### 2.10. Statistical Analysis

The Kolmogorov-Smirnov test was used to verify data normality. Subsequently, the data that did not pass the test were analyzed by a Kruskal-Wallis test. The comparison between days 0 and 15 in FST was assessed by an unpaired Student *t*-test, while the comparison between multiple groups was performed with a two-way ANOVA followed by a Holm-Sidak multiple comparison test. Post hoc comparisons were only followed after main factors showed statistical significance; main factors used were CUS with two levels (present or absent (control)) and pharmacological treatment with three levels (vehicle, URB597, and RIM+URB597). A *p* < 0.05 was considered statistically significant, and we performed the analyses on GraphPad Prism 8 software.

## 3. Results

### 3.1. Corticosterone Serum Levels

**Figure 2:**
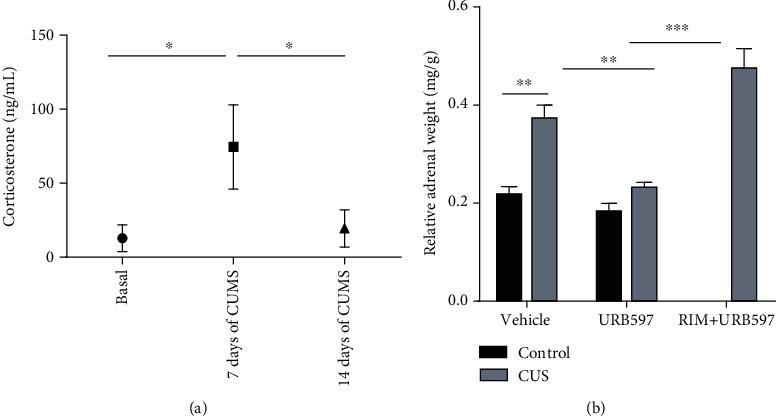
(a) Results of serum corticosterone concentrations at days 0, 7, and 14 of CUS exposure. The columns represent the mean ± SEM, *n* = 5/group. ^∗^Statistically significant differences, *p* ≤ 0.05; Kruskal-Wallis. (b) The right graph shows the relative weights of the adrenal glands at the end of the experiment. The columns represent the mean ± SEM, *n* = 6/group; ^∗∗^*p* ≤ 0.01; ^∗∗∗^*p* ≤ 0.001. Two-way ANOVA post hoc Holm-Sidak's.

To assess CUS effects upon glucocorticoid levels, an ELISA test was performed to detect the most abundant glucocorticoid in mice, corticosterone. Serum corticosterone concentrations were obtained in different cohorts during the CUS model at 0, 7, and 14 days. Statistical analysis showed a significant increase in corticosterone concentration at day 7 (74 ± 14 ng/mL) compared to day 0 without stress (12 ± 4 ng/mL, *p* = 0.007). This increase was absent in the mice after 14 days of stress (19 ± 6 ng/mL) (Figure 2(a)).

### 3.2. Adrenal Gland Relative Weight

The CUS model generated changes in the adrenal glands. Relative weight of adrenal glands was quantified as gland weight in milligram/body weight in grams at the end of the experimental design. The 2-way ANOVA showed significance for the interaction: *F*_2,27_: 20.90, *p* = 0.0001; factor treatment: *F*_2,27_: 7.72, *p* = 0.0027; and factor CUS *F*_1,27_: 84.24, *p* = 0.0001. Our results show a statistically significant increase (*p* = 0.008) in the relative weight of adrenal glands in animals undergoing CUS compared to the control group. Furthermore, treatment with URB597 after CUS (CUS+URB597) caused a statistically significant decrease (*p* = 0.0012) in the relative weight of adrenal glands compared to the CUS group. Interestingly enough, this effect induced by URB957 was significantly reversed by the prior administration of the CB1R antagonist RIM. Due to a significant increase (*p* = 0.0001) in the weight of the adrenal glands that was observed in the CUS+RIM+URB597 group compared to the CUS+URB group, it is worth mentioning that in animals treated only with URB597, the weight of the adrenal glands did not change compared to control group (Figure 2(b)).

### 3.3. TBARS Assay

**Figure 3:**
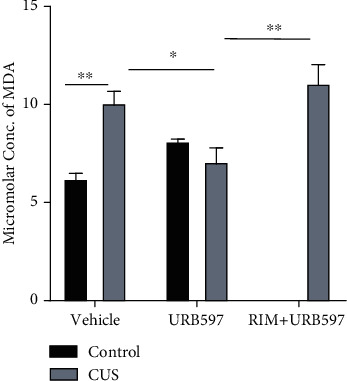
TBARS assay results. Columns represent mean of MDA concentration ± SEM, *n* = 6/group; ^∗^*p* ≤ 0.05; ^∗∗^*p* ≤ 0.01. Two-way ANOVA post hoc Holm-Sidak's.

The 2-way ANOVA for this assay showed significance for the interaction: *F*_2,27_: 25.95, *p* = 0.0001; factor treatment: *F*_2,27_: 4.90, *p* = 0.0156; and factor CUS *F*_1,27_: 50.45, *p* = 0.0001. Mice that underwent the CUS model had shown a significant augmentation of MDA in the hippocampus when compared to the control group (*p* = 0.0043) (see Figure 3). These effects of CUS were reverted by the inhibition of FAAH by URB597 administration; hence, the CUS+URB597 group showed a diminishment of MDA levels vs. CUS alone (*p* = 0.0342) in this region. Finally, in this trial, when the antagonist for CB1R was administered, levels of MDA raised again (CUS+RIM+URB597 vs. CUS+URB597) significantly (*p* = 0.0033).

### 3.4. Forced Swimming Test

**Figure 4:**
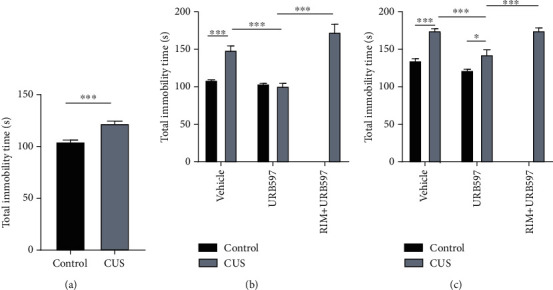
Total immobility time (s) in FST. (a) Evaluations at days 0 and 15, representing control and CUS conditions, ^∗∗∗^*p* ≤ 0.001. Unpaired Student's *t*-test. Evaluations at day 22 (b) and day 28 (c) under different experimental treatment conditions. The columns represent the mean of total immobility time ± SEM, *n* = 12/group; ^∗^*p* ≤ 0.05; ^∗∗∗^*p* ≤ 0.001. Two-way ANOVA post hoc Holm-Sidak's.

Chronically stressed animals exhibited a significant increase in immobility time compared to control animals. Immobility time in seconds was significantly greater (*p* = 0.001) in FST on day 15 after the CUS model compared to the control test on day 0 (Figure 4(a)). The 2-way ANOVA for the total immobility time at day 22 showed significance for the interaction: *F*_2,53_: 25.52, *p* = 0.0001; factor treatment: *F*_2,53_: 11.18, *p* = 0.0001; and factor CUS *F*_1,53_: 61.42, *p* = 0.0001. The 2-way ANOVA for total immobility time at day 29 showed significance for the interaction: *F*_2,53_: 30.29, *p* = 0.0001; factor treatment: *F*_2,53_: 27.12, *p* = 0.0001; and factor CUS *F*_1,53_: 125.6, *p* = 0.0001. The control group maintained significant differences against the CUS group in both the test on day 22 (*p* = 0.0003) and the test on day 29 (*p* = 0.0001). For his part, the group treated with FAAH inhibitor (CUS+URB597) showed significant differences against the CUS group in both the test on day 22 (*p* = 0.0001) and the test on day 29 (*p* = 0.0003). On the other hand, the effects caused by URB597 after CUS were blocked in the group of animals that received administration of RIM (CUS+RIM+URB597), in which a significant increase in time of immobility was observed on day 22 compared to the CUS+URB597 group (*p* = 0.0001). These effects were maintained until day 29 (*p* = 0.0003) (Figures 4(b) and 4(c)). Particularly, significant differences between URB597 and CUS+URB597 groups were observed on day 29 for this experiment (*p* = 0.024).

### 3.5. Splash Test

**Figure 5:**
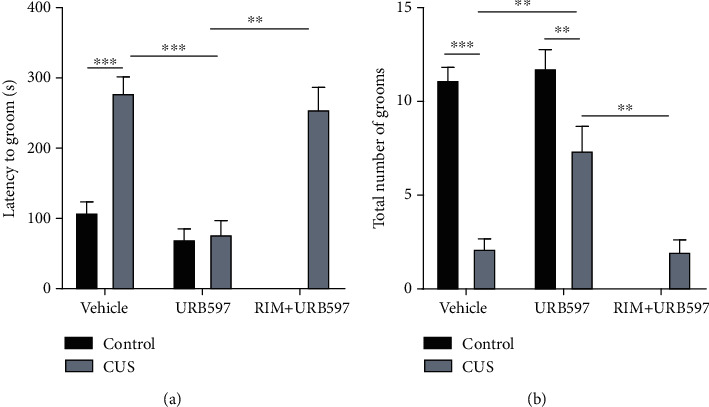
(a) Latency time to first grooming. (b) Total frequency of grooming. Columns represent mean ± SEM, *n* = 12/group; ^∗∗^*p* ≤ 0.01; ^∗∗∗^*p* ≤ 0.001. Two-way ANOVA post hoc Holm-Sidak's.

The 2-way ANOVA for the latency to groom showed significance for the interaction: *F*_2,55_: 11.22, *p* = 0.0009; factor treatment: *F*_2,55_: 15.58, *p* = 0.0002; and factor CUS *F*_1,55_: 42.30, *p* = 0.0001. The 2-way ANOVA for the total number of grooms showed significance for the interaction: *F*_2,55_: 9.45, *p* = 0.0012; factor treatment: *F*_2,55_: 20.61, *p* = 0.0001; and factor CUS *F*_1,55_: 14.69, *p* = 0.0010. Data presented herein (Figure 5) demonstrated that animals subjected to the CUS protocol neglected coat grooming when compared to the control group. This was illustrated by increased latency to start the first grooming (*p* = 0.0003) and decreased total number (*p* = 0.0001) of grooms. Animals treated with URB597 after CUS (CUS+URB597) showed a decrease in latency to start the first grooming (*p* = 0.0003) and an increase (*p* = 0.0047) in the total number of grooms, compared to the group undergoing CUS alone. Interestingly enough, these effects induced by URB957 were significantly reversed by the prior administration of the CB1R antagonist RIM. The CUS+RIM+URB597 group presented significant differences in latency time (*p* = 0.0013) and in the total number of grooms (*p* = 0.0081), respectively, against the CUS+URB597 group. Particularly, a significant difference between URB597 and CUS+URB597 groups was observed on the total number of grooms for this experiment (*p* = 0.0239).

### 3.6. BDNF Expression

**Figure 6:**
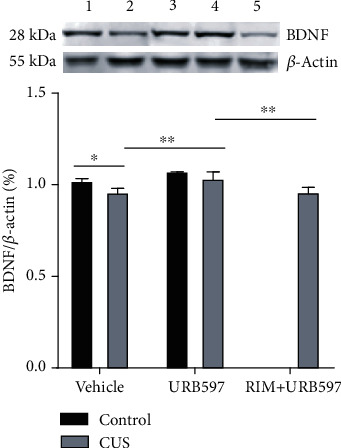
BDNF expression in the hippocampus. 1, control; 2, CUS; 3, CUS+URB597; 4, URB597; 5, CUS+RIM+URB597. Columns represent average of relative intensity of BDNF to *β* − actin ± SEM, *n* = 6/group; ^∗^*p* ≤ 0.05; ^∗∗^*p* ≤ 0.01. Two-way ANOVA post hoc Holm-Sidak's.

The protein expression of BDNF in the hippocampus of the different groups is presented in Figure 6. The 2-way ANOVA showed significance for the interaction: *F*_2,27_: 389.8, *p* = 0.0001; factor treatment: *F*_2,27_: 452.3, *p* = 0.0001; and factor CUS *F*_1,27_: 397.1, *p* = 0.0001. The levels of BDNF protein in the hippocampus were significantly decreased in the CUS group vs. the control group (*p* = 0.0178). Chronic URB597 (CUS+URB597) treatment elevated BDNF expression in the hippocampus, compared to the group undergoing CUS alone (*p* = 0.002). On the other hand, the effects caused by URB597 after CUS were blocked in the group of animals that received administration of RIM (CUS+RIM+URB597), respectively, against the CUS+URB597 group (*p* = 0.0015).

### 3.7. BrdU/Sox2 Double Fluorescent Immunohistochemistry

**Figure 7:**
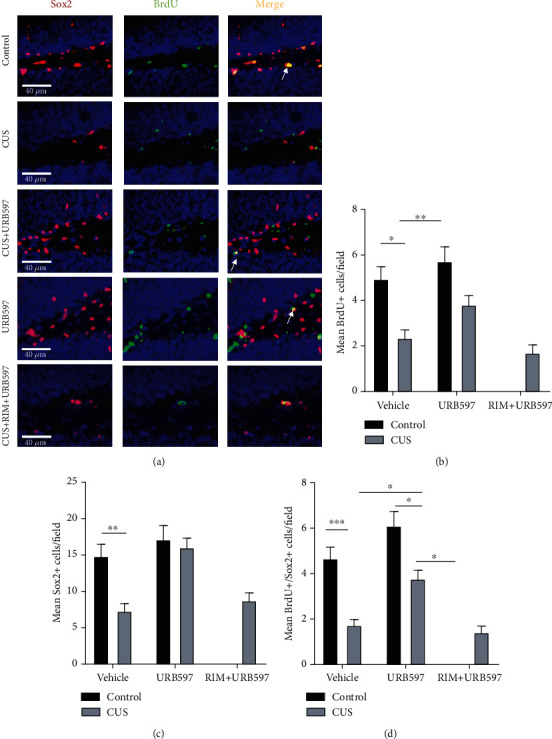
(a) Representative photomicrographs of the subgranular zone of the hippocampus where BrdU+/Sox2+ marking is observed. Marking for BrdU+ is seen in green, and the marking in red corresponds to Sox2+. The last panel of photomicrographs corresponds to the colocation of the two markings. Arrows indicate the positive cells analyzed. 40x bar = 40 *μ*m. (b) Average of Sox2-positive cells per field of DG in the hippocampus. (c) Average of BrdU-positive cells per field in the same region. (d) Mean number of merged cells per field. Columns represent the average of the cells counted in each field ± SEM, *n* = 6/group; ^∗^*p* ≤ 0.05; ^∗∗^*p* ≤ 0.01; ^∗∗∗^*p* ≤ 0.001. Two-way ANOVA post hoc Holm-Sidak's.

To label proliferative cells, we injected 100 mg/kg of BrdU two hours before sacrifice (Figure 7(a)). The 2-way ANOVA for the Sox2-positive cells per field showed significance for the interaction: *F*_2,27_: 3.53, *p* = 0.0337, and factor treatment: *F*_2,27_: 9.30, *p* = 0.0002, and not differences for the factor CUS alone *F*_1,27_: 0.02, *p* = 0.9875. Our data indicated that the number of Sox2 cells in the CUS group was significantly lesser as compared with the control group (*p* = 0.0215). Interestingly, we can also observe that the group CUS+URB597 shows a significant increase in these positive cells compared to the CUS group (*p* = 0.0046) (Figure 7(b)). For his part, the 2-way ANOVA for the BrdU-positive cells per field showed significance for the interaction: *F*_2,27_: 6.78, *p* = 0.0019, and factor treatment: *F*_2,27_: 20.86, *p* = 0.0001, and not differences for the factor CUS alone *F*_1,27_: 4.00, *p* = 0.0485. These results indicated that the number of BrdU cells in the CUS group was significantly lesser when compared with that in the control group (*p* = 0.0028) (Figure 7(c)). Finally, the 2-way ANOVA for the BrdU-/Sox2-positive cells per field showed significance for the interaction: *F*_2,27_: 3.19, *p* = 0.0463, and factor treatment: *F*_2,27_: 14.81, *p* = 0.0001, and not differences for the factor CUS alone *F*_1,27_: 4.30, *p* = 0.0412. The results for this double labeling are similar to the single counts abovementioned but have more differences. Double-positive cells in the CUS group were significantly lesser as compared with those in the control group (*p* = 0.0006). Interestingly, we can also observe that the group CUS+URB597 shows a significant increase in these positive cells compared to the CUS group (*p* = 0.0266). However, the effect induced by URB597 was reversed in the group administered with the CB1R antagonist CUS+RIM+URB597 compared with the CUS+URB597 group (*p* = 0.0266) (Figure 7(d)). Particularly, significant differences between URB597 and CUS+URB597 groups were observed only on the amount of double-positive cells per field for this experiment (*p* = 0.0098).

## 4. Discussion

In the present study, exposure to CUS induces depressive-like behavior in mice, as well as a significant decrease in the expression of primary neuronal precursors of the subgranular zone of DG in the hippocampus. Damage caused by stress is a consequence of different biochemical alterations that alter brain homeostasis, the whole nervous system, and therefore the behavior of individuals, causing an increase in oxidative stress, proinflammatory processes, depletion of mitochondrial function, and alterations in the mechanisms of cellular signaling, among others [33]. To mitigate these effects, there are different pharmacological approaches; however, one of the most innovative is the stimulation of the endocannabinoid system. To our knowledge, this is the first study to investigate the neuroprotective effect that inhibition of the FAAH enzyme can exert by chronic administration of URB97 in a murine model of CUS. The CUS model has been employed as a tool for the study of the neurobiology of depression. Its validity relies not only on the face criteria (which is based on the generation of depressive-like behaviors) but also on the predictability and construct validity. These lastly mentioned criteria are related to the model response to actually approved treatments (and therefore the predictability for assessment of new drugs) and with the physiological, molecular, and morphological biomarkers and other objectively measurable features seen in the clinic (construct validity), respectively. Its benefits are a general approach to an *in vivo* model for depression that is not related to genetics and therefore gives more information about the environmental causes of the condition, while a limitation of the model is that the translation to the clinical use is at least complicated due the differences between mice and humans [21, 22].

### 4.1. Effect of URB597 on Biomarkers in the CUS Model

Given the close relationship that exists between long-term stress and depressive behavior, we decided to investigate corticosteroid levels as one of the main biomarkers of stress; likewise, to obtain more information, we analyzed the relative weight of the adrenal glands. Corticosterone concentration was determined at baseline and on days 7 and 14 of CUS exposure. Our results show that the exposure of animals to CUS generates a significant increase in corticosterone levels on day 7 of exposure and that these values decrease on day 14. The elevation of corticosterone levels is consistent with other author's results in similar models [2, 34, 35], although the measurements in these studies were only at the end of treatments. On the other hand, an investigation carried out by Gong et al. [4], with the CUS model, describes a sustained increase in corticosterone from days 2 to 8 of exposure. In this investigation, a group of animals subjected to stress was also carried out using the movement restriction model (single stressor); interestingly, corticosterone in this group peaked on day 2 and progressively decreased, suggesting that CUS could keep corticosterone levels elevated longer due to a lower degree of habituation. Models of acute stressors such as sleep deprivation have also obtained an increase in corticosterone that is not maintained, in animals with a long time of deprivation [34]. There are 2 possible explanations for the increase of corticosterone and a subsequent decrease of this molecule under CUS that we find. The first implies negative feedback on the HPA axis that remains even under the hyperactivity that is present after CUS [36] and second, a decompensation where the adrenal cortex cannot meet the demand for glucocorticoids and it decreases even though there is a high concentration of the hormone adrenocorticotropin (ACTH) [37]. Other pieces of evidence propose the measurement of the relative weight of the adrenal glands as an indicator of dysregulation of the HPA axis in various models of depression [32]. Animals subjected to a CUS protocol exhibit increased levels of corticosterone and an increase in adrenal gland weight [38]. An increase in the relative weight of these glands may be indicative of the increase in maximum corticosterone response to ACTH [39]. Our results show an increase in the relative weight of adrenal glands for the group with the CUS model, which is consistent with other investigations [2, 40]. Interestingly, we found the relative weight of the group administered with CUS+URB597 lower compared to that with CUS. In this sense, adrenocortical steroidogenesis within the human adrenal is directly influenced by the endocannabinoid system via CB1R. Hillard and collaborators [41] have discussed that endocannabinoid tone negatively modulates HPA axis activity. It is suggested that upon exposure to stress, endocannabinoid levels rapidly decline through an undetermined mechanism, resulting in a disinhibition of glutamatergic projections to the PVN and allowing activation of the hypothalamus [42]. Work carried out by Ziegler and collaborators [37] demonstrated that the cannabinoid receptors CB1R and CB2R are expressed in the adrenal gland, and the activation of these receptors with anandamide inhibits the release of corticosterone. Therefore, a decrease in the weight of the adrenal gland may indicate an inhibitory action over different parts of the HPA axis by the cannabinoid system and vice versa [43]. A direct effect of stress hormones, like glucocorticoids, in the induction of the brain oxidative damage has been shown [44] as induced oxidative load in the brain with a significant increase in prooxidant (lipid peroxidation and nitrite levels) markers and a substantial decline in antioxidant defense (catalase and reduced glutathione levels) system. Nowadays, it is well known that oxidative stress and therefore lipid peroxidation are present in depressed patients and those more susceptible to suffer these disorders, like elder individuals [45, 46]. As part of the CUS model, different oxidative features have been reported such as higher MDA and reactive oxygen species. As a part of our results, the inhibition of FAAH by URB597 was able to block the increase in MDA concentration. Although the URB597 molecule per se has an antioxidant effect on his structure and has been proposed as a modulator of lipid mediators recently [47], the most probable and well-described mechanism by which it can exert antioxidant properties is by promoting NRF2 protein activity. This transcription factor is responsible for the biosynthesis of cytoprotective antioxidant proteins hemoxigenase-1 (HO-1), n-quinone oxidase (NQO1), and glutamate-cysteine ligase (GCLc). All these pieces of evidence make a convincing explanation of the antioxidant advantages of this pharmacological strategy [48].

### 4.2. Effect of URB597 on Behavioral Tests and BDNF Implications

The results of the present work establish that the chronic administration of URB597 elicited an antidepressant-like behavior in the forced swim (hopelessness) and splash tests (anhedonia). Preclinical studies have shown that pharmacological blockade of CB1R rendered animals more emotionally reactive and anxious [49, 50], susceptible to chronic stress-induced anhedonia [50], and to even manifest a depressive phenotype [51] as well as being liable to impairments in HPA axis regulation [52] reminiscent of neuroendocrine dysfunction observed in depression. Antidepressant-like effects in the FST have also been reported previously with the endocannabinoid reuptake inhibitor AM404 [N-(4-hydroxyphenyl)-arachidonamide] and the direct CB1R agonist HU-210 [3-(1,1-dimethyl heptyl)-()-11-hydroxy-8-tetrahydro-cannabinol] [53], although this is the first study to our knowledge that evaluates this test in a repeated paradigm. Antidepressant-like activity from the selective FAAH inhibitor URB597 [cyclohexylcarbamic acid 3-carbamoyl biphenyl-3yl ester] has been also demonstrated in the tail suspension test [54, 55] and chronic mild stress paradigms [56, 57]. Along with the results shown in the present article, these pieces of evidence support the concept that the endocannabinoid system activation through its endogenous ligands may serve as a target for depression therapy [58, 59]. In addition, works carried out by Bambico and collaborators [60] suggest that FAAH genetic deletion enhances anxiolytic-like and antidepressant-like effects, with an enhancement in the spontaneous activity of neurons from the dorsal raphe, through an increase in the firing rate of serotonergic neurons [60]. This could increase participation of the serotoninergic system, which is impaired during depressive disorders, thus explaining the effects of this molecule on the diminishment of depressive-like behaviors. Our findings show statistical differences between groups URB597 and CUS+URB597 for the behavioral tests; this suggests that other non-AEA/CB1R-mediated mechanisms could be participating such as the serotoninergic system aforementioned. Nevertheless, that is not the only explanation for these results; it also has been shown that neurotrophins play an important role in the modulation of depressive behaviors. Previous reports show that low levels of BDNF in the hippocampus may lead to some functional and structural alterations in hippocampal neurons, induce depressive-like behaviors in rodents, and ultimately contribute to the symptoms of depression in humans [61]. Consistent with these observations, previous studies have shown that CB1R (-/-) knockout mice exhibit an augmented response to stress (increased despair behavior and corticosterone) with decreased BDNF levels in the hippocampus [62]. Notably, local administration of BDNF in the hippocampus reversed the increased despair behavior of CB1R (-/-) knockout mice. Although the role of BDNF in depressive behavior is yet to be clearly understood, the potential role of BDNF in neuronal plasticity, dendrite development, and modulation of depressive behaviors makes it a reliable therapeutic target in the treatment of depressive disorders [63, 64]. Activation of the tyrosine kinase B receptor (TrkB) by BDNF has been proven to play a critical role in synaptic plasticity mechanisms, as well as synaptic efficacy [65]. In this sense, as listed in our results, we could observe a CUS-induced decrease in BDNF levels accompanied by depressive-like behavior. As we expected from previous reports from Vinod and collaborators [19] who saw an increase in the levels of this neurotrophin in Kyoto rats, herein, the decrease in BDNF was reversed when the enzyme FAAH was inhibited, an effect probably mediated through the activation of the CB1R. It remains to be seen if the endocannabinoid system-mediated BDNF function promotes neuronal plasticity leading to attenuation of depressive-like behavior. Some other reports suggested that cannabinoids appear to elicit antidepressant-like effects through the promotion of hippocampal neurogenesis [66]. Hippocampal cell proliferation is a downstream sequela of antidepressant treatment [67], which is why we also assessed neural progenitors with BrdU/Sox2 colabeling.

### 4.3. Effects of URB597 on BrdU/Sox2 Marking

Various events have been reported that may influence the proliferation and survival of neural precursors in the subgranular zone of the hippocampus. In this sense, stress has been shown to negatively affect this process [61, 68]. For example, patients with depression exhibit decreased levels of neurogenesis [69, 70]; also, neurogenesis ablation increases innate anxiety-like behaviors [71] and depressive-like symptoms [72] in animal models. And more importantly, antidepressant drugs increase neurogenesis, an effect that is required to observe some of its behavioral effects in rodents [73, 74]. One transcription factor which influences this process is Sox2, which is elemental for the process of newborn cells, as *in vitro* and *in vivo* studies show [75, 76]. One of the roles that this protein plays in the brain is to maintain the identity of the neural precursors [77, 78], and therefore, it is considered a marker of neural progenitors and stem cells [79]. Numerous investigations have demonstrated its participation in the neurogenic process [80]; elevated levels of the Sox2 protein have been reported in patients undergoing treatment for the depressive state [81]. We were able to find that our model generated a lack of this protein, whereas the inhibition of FAAH was able to restore the levels of this molecule unless antagonism of CB1R was provoked. Regarding this, the endocannabinoid system is a key regulator in the generation, survival, maturation, and functional integration of neuronal genesis in the adult hippocampus. Neural progenitor cells and their descendants express a functional endocannabinoid system and are subject to the effects of endocannabinoid signaling [82]. Activation of CB1R induces neuronal proliferation, maintenance, and differentiation in DG [16], which is attenuated in mice lacking CB1R (-/-) [83, 84]. In addition, various intracellular signaling pathways regulated by the endocannabinoid system converge mainly on the Akt/mTOR and MAPK/CREB pathways, which are critically involved in cell proliferation, differentiation, and survival and are necessary for endocannabinoids to exert their proneurogenic effects [82]. The elimination of the enzyme responsible for the hydrolysis of AEA, FAAH, increases cell proliferation in the DG of adult mice [85]. These findings illustrate the importance of increasing endocannabinoid tone to maintain neurogenesis in the event of stress. Despite this, differences between URB597 and CUS+URB597 groups were found on the neural precursors marked, pointing out the possible combination with other known effectors to have a better response against stressful conditions. This work suggests, that blockade of all the CUS-related effects in the CUS+URB597 group were probably mediated by CB1R activation, reached by the augmentation of AEA levels after URB597 administration. Nevertheless, the lack of a RIM-control group represents a limitation of this study that implies the impossibility of the authors to prove a CB1R-mediated effect. This is especially important to address, given the facts that the recovery of the CUS effect after administration of RIM could be due to other, CUS-unrelated CB1R effects overriding the influence that URB had on AEA/CB1R signaling and that AEA can bind other non-CB1R substrates also implicated in stress-regulation responses such as CB2R.

## 5. Conclusions

Our overall results suggest that inhibition of FAAH was able to reverse the depressive-like behaviors generated in mice after the model. Likewise, other physiological responses associated with CUS were reduced in the treated group, among them, an increase in the relative weight of the adrenal glands and lipid oxidation and decreased BDNF levels and the number of neural precursors. These favorable responses to enzyme inhibitor administration were blocked by employing CB1R antagonist RIM. Chronic administration of URB597 generated an antidepressant overall effect on mice under the CUS model. These results encourage us to keep investigating this pharmacological strategy to determine its full potential.

## Data Availability

The data used to support the findings of this study are available from the corresponding author upon request.
